# 10-Hexyl-10*H*-phenothia­zine-3-carbaldehyde

**DOI:** 10.1107/S1600536808038890

**Published:** 2008-11-26

**Authors:** Hongli Wang, Wenyuan Xu, Bin Zhang, Wenjing Xiao, Hong Wu

**Affiliations:** a Department of Chemistry, Central China Normal University, Wuhan, Hubei 430079, People’s Republic of China

## Abstract

The asymmetric unit of the title compound, C_19_H_21_NOS, contains two mol­ecules, which form dimers *via* pairs of weak C—H⋯O hydrogen bonds.

## Related literature

For the synthesis, see: Krishna *et al.* (1999 ▶). For general background, see: Hauck *et al.* (2007 ▶).
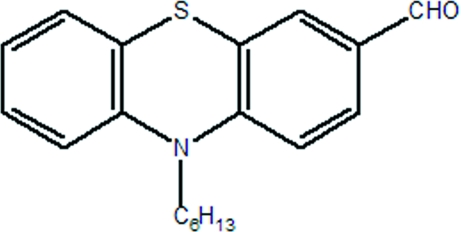


## Experimental

### 

#### Crystal data

C_19_H_21_NOS
*M* *_r_* = 311.43
Triclinic, 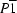

*a* = 8.4073 (9) Å
*b* = 13.7719 (15) Å
*c* = 14.6485 (15) Å
α = 93.957 (2)°
β = 98.781 (2)°
γ = 90.983 (2)°
*V* = 1671.5 (3) Å^3^
*Z* = 4
Mo *K*α radiation
μ = 0.20 mm^−1^
*T* = 298 (2) K
0.23 × 0.20 × 0.10 mm

#### Data collection

Bruker SMART CCD diffractometer
Absorption correction: multi-scan (*SADABS*; Sheldrick, 1997 ▶) *T*_min_ = 0.957, *T*_max_ = 0.981
9878 measured reflections
5800 independent reflections
3575 reflections with *I* > 2σ(*I*)
*R*_int_ = 0.089

#### Refinement

*R*[*F* ^2^ > 2σ(*F* ^2^)] = 0.079
*wR*(*F* ^2^) = 0.210
*S* = 0.96
5800 reflections
399 parameters
H-atom parameters constrained
Δρ_max_ = 0.35 e Å^−3^
Δρ_min_ = −0.27 e Å^−3^

### 

Data collection: *SMART* (Bruker, 2000 ▶); cell refinement: *SAINT* (Bruker, 2000 ▶)’; data reduction: *SAINT*; program(s) used to solve structure: *SHELXS97* (Sheldrick, 2008 ▶); program(s) used to refine structure: *SHELXL97* (Sheldrick, 2008 ▶); molecular graphics: *SHELXTL* (Sheldrick, 2008 ▶); software used to prepare material for publication: *SHELXTL*.

## Supplementary Material

Crystal structure: contains datablocks global, I. DOI: 10.1107/S1600536808038890/hb2847sup1.cif

Structure factors: contains datablocks I. DOI: 10.1107/S1600536808038890/hb2847Isup2.hkl

Additional supplementary materials:  crystallographic information; 3D view; checkCIF report

## Figures and Tables

**Table 1:** Hydrogen-bond geometry (Å, °)

*D*—H⋯*A*	*D*—H	H⋯*A*	*D*⋯*A*	*D*—H⋯*A*
C28—H28⋯O1	0.93	2.54	3.454 (5)	168
C9—H9⋯O2	0.93	2.50	3.394 (5)	162

**Fig. 1.**
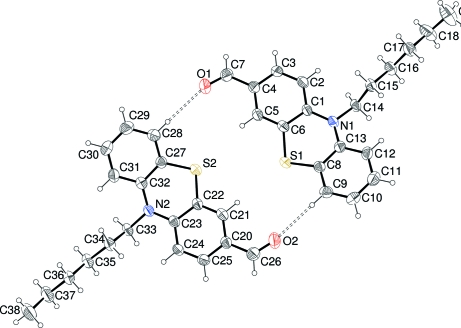
The molecular structure of (I) showing 50% displacement ellipsoids for the non-hydrogen atoms. The hydrogen bonds are indicated by dashed lines.

## supplementary crystallographic information

### Comment

We used the Vilsmeier reaction to obtain the title compound, (I), which is a good intermediate for several compounds (Hauck *et al.*, 2007).

The asymmetric unit of (I) contains two molecules (Fig. 1), which form dimers via pairs of weak C—H···O bonds (Table 1).

### Experimental

The title material, prepared by a literature method (Krishna *et al.* 1999), was collected by filtration and recrystallized from chloroform as yellow blocks of (I).

### Refinement

The H atoms were geometrically placed (C—H = 0.93-0.97Å) and refined as riding with *U*_iso_(H) = 1.2*U*_eq_(C) or 1.5*U*_eq_(methyl C).

### Crystal data

**Table:**

C_19_H_21_NOS	*Z* = 4
*M**_r_* = 311.43	*F*_000_ = 664
Triclinic, *P*1	*D*_x_ = 1.238 Mg m^−^^3^
Hall symbol: -P 1	Mo *K*α radiation λ = 0.71073 Å
*a* = 8.4073 (9) Å	Cell parameters from 2010 reflections
*b* = 13.7719 (15) Å	θ = 2.5–21.1º
*c* = 14.6485 (15) Å	µ = 0.20 mm^−^^1^
α = 93.957 (2)º	*T* = 298 (2) K
β = 98.781 (2)º	Block, yellow
γ = 90.983 (2)º	0.23 × 0.20 × 0.10 mm
*V* = 1671.5 (3) Å^3^	

### Data collection

**Table:**

Bruker SMART CCD diffractometer	5800 independent reflections
Radiation source: fine-focus sealed tube	3575 reflections with *I* > 2σ(*I*)
Monochromator: graphite	*R*_int_ = 0.089
*T* = 298(2) K	θ_max_ = 25.0º
ω scans	θ_min_ = 1.5º
Absorption correction: multi-scan(SADABS; Sheldrick, 1997)	*h* = −9→9
*T*_min_ = 0.957, *T*_max_ = 0.981	*k* = −16→16
9878 measured reflections	*l* = −17→10

### Refinement

**Table:**

Refinement on *F*^2^	Secondary atom site location: difference Fourier map
Least-squares matrix: full	Hydrogen site location: inferred from neighbouring sites
*R*[*F*^2^ > 2σ(*F*^2^)] = 0.079	H-atom parameters constrained
*wR*(*F*^2^) = 0.210	*w* = 1/[σ^2^(*F*_o_^2^) + (0.0951*P*)^2^ + 0.4339*P*] where *P* = (*F*_o_^2^ + 2*F*_c_^2^)/3
*S* = 0.96	(Δ/σ)_max_ = 0.008
5800 reflections	Δρ_max_ = 0.35 e Å^−^^3^
399 parameters	Δρ_min_ = −0.26 e Å^−^^3^
Primary atom site location: structure-invariant direct methods	Extinction correction: none

### Special details

**Table:**

Geometry. All e.s.d.'s (except the e.s.d. in the dihedral angle between two l.s. planes) are estimated using the full covariance matrix. The cell e.s.d.'s are taken into account individually in the estimation of e.s.d.'s in distances, angles and torsion angles; correlations between e.s.d.'s in cell parameters are only used when they are defined by crystal symmetry. An approximate (isotropic) treatment of cell e.s.d.'s is used for estimating e.s.d.'s involving l.s. planes.
Refinement. Refinement of *F*^2^ against ALL reflections. The weighted *R*-factor *wR* and goodness of fit *S* are based on *F*^2^, conventional *R*-factors *R* are based on *F*, with *F* set to zero for negative *F*^2^. The threshold expression of *F*^2^ > σ(*F*^2^) is used only for calculating *R*-factors(gt) *etc*. and is not relevant to the choice of reflections for refinement. *R*-factors based on *F*^2^ are statistically about twice as large as those based on *F*, and *R*- factors based on ALL data will be even larger.

### Fractional atomic coordinates and isotropic or equivalent isotropic displacement parameters (Å^2^)

**Table:**

	*x*	*y*	*z*	*U*_iso_*/*U*_eq_	
C1	0.8046 (4)	−0.0018 (3)	1.0692 (3)	0.0498 (9)	
C2	0.7545 (5)	−0.0942 (3)	1.0280 (3)	0.0614 (11)	
H2	0.7849	−0.1490	1.0594	0.074*	
C3	0.6625 (5)	−0.1058 (3)	0.9428 (3)	0.0633 (11)	
H3	0.6285	−0.1681	0.9183	0.076*	
C4	0.6185 (5)	−0.0276 (3)	0.8922 (3)	0.0575 (10)	
C5	0.6693 (5)	0.0642 (3)	0.9305 (3)	0.0562 (10)	
H5	0.6431	0.1179	0.8965	0.067*	
C6	0.7571 (5)	0.0781 (3)	1.0172 (3)	0.0556 (10)	
C7	0.5184 (5)	−0.0421 (3)	0.8011 (3)	0.0695 (12)	
H7	0.4809	−0.1052	0.7820	0.083*	
C8	0.9599 (5)	0.1833 (3)	1.1502 (3)	0.0537 (10)	
C9	1.0577 (5)	0.2649 (3)	1.1820 (3)	0.0638 (11)	
H9	1.0396	0.3227	1.1532	0.077*	
C10	1.1795 (6)	0.2617 (3)	1.2545 (3)	0.0707 (12)	
H10	1.2427	0.3170	1.2764	0.085*	
C11	1.2069 (5)	0.1751 (4)	1.2945 (3)	0.0737 (12)	
H11	1.2896	0.1721	1.3442	0.088*	
C12	1.1154 (5)	0.0931 (3)	1.2629 (3)	0.0624 (11)	
H12	1.1386	0.0352	1.2907	0.075*	
C13	0.9881 (5)	0.0945 (3)	1.1901 (3)	0.0518 (9)	
C14	0.8990 (5)	−0.0712 (3)	1.2169 (3)	0.0569 (10)	
H14A	0.7963	−0.1065	1.2038	0.068*	
H14B	0.9123	−0.0455	1.2810	0.068*	
C15	1.0314 (5)	−0.1438 (3)	1.2071 (3)	0.0620 (11)	
H15A	1.0262	−0.1668	1.1426	0.074*	
H15B	1.1359	−0.1123	1.2279	0.074*	
C16	1.0099 (5)	−0.2288 (3)	1.2645 (3)	0.0658 (11)	
H16A	1.0017	−0.2033	1.3270	0.079*	
H16B	0.9084	−0.2621	1.2394	0.079*	
C17	1.1389 (5)	−0.3018 (3)	1.2700 (4)	0.0755 (13)	
H17A	1.2404	−0.2697	1.2971	0.091*	
H17B	1.1493	−0.3269	1.2077	0.091*	
C18	1.1080 (7)	−0.3864 (4)	1.3264 (5)	0.112 (2)	
H18A	1.0881	−0.3594	1.3864	0.134*	
H18B	1.0090	−0.4194	1.2963	0.134*	
C19	1.2236 (9)	−0.4567 (5)	1.3419 (6)	0.152 (3)	
H19A	1.2557	−0.4793	1.2842	0.229*	
H19B	1.1795	−0.5104	1.3693	0.229*	
H19C	1.3155	−0.4291	1.3831	0.229*	
C20	0.7245 (5)	0.5290 (3)	0.9780 (3)	0.0549 (10)	
C21	0.6665 (5)	0.4380 (3)	0.9407 (3)	0.0553 (10)	
H21	0.7115	0.3831	0.9666	0.066*	
C22	0.5447 (4)	0.4260 (3)	0.8668 (3)	0.0509 (9)	
C23	0.4783 (4)	0.5078 (3)	0.8231 (3)	0.0511 (9)	
C24	0.5365 (5)	0.5994 (3)	0.8624 (3)	0.0593 (10)	
H24	0.4936	0.6550	0.8368	0.071*	
C25	0.6555 (5)	0.6090 (3)	0.9380 (3)	0.0617 (11)	
H25	0.6906	0.6710	0.9629	0.074*	
C26	0.8506 (6)	0.5412 (3)	1.0586 (3)	0.0705 (12)	
H26	0.8861	0.6044	1.0792	0.085*	
C27	0.3997 (4)	0.3210 (3)	0.7143 (3)	0.0537 (9)	
C28	0.3913 (5)	0.2390 (3)	0.6537 (3)	0.0658 (11)	
H28	0.4292	0.1805	0.6754	0.079*	
C29	0.3283 (6)	0.2428 (4)	0.5627 (3)	0.0823 (14)	
H29	0.3215	0.1872	0.5225	0.099*	
C30	0.2749 (6)	0.3302 (4)	0.5313 (3)	0.0829 (14)	
H30	0.2294	0.3329	0.4695	0.099*	
C31	0.2877 (5)	0.4140 (3)	0.5896 (3)	0.0708 (12)	
H31	0.2549	0.4728	0.5664	0.085*	
C32	0.3497 (4)	0.4110 (3)	0.6834 (3)	0.0548 (10)	
C33	0.2671 (5)	0.5804 (3)	0.7159 (3)	0.0574 (10)	
H33A	0.2457	0.6174	0.7713	0.069*	
H33B	0.1641	0.5570	0.6817	0.069*	
C34	0.3427 (5)	0.6498 (3)	0.6567 (3)	0.0663 (11)	
H34A	0.4403	0.6796	0.6921	0.080*	
H34B	0.3716	0.6135	0.6028	0.080*	
C35	0.2280 (5)	0.7279 (3)	0.6258 (3)	0.0703 (12)	
H35A	0.1947	0.7608	0.6802	0.084*	
H35B	0.1327	0.6972	0.5886	0.084*	
C36	0.2949 (5)	0.8034 (3)	0.5705 (3)	0.0719 (12)	
H36A	0.3906	0.8338	0.6074	0.086*	
H36B	0.3271	0.7707	0.5157	0.086*	
C37	0.1802 (7)	0.8810 (4)	0.5411 (4)	0.0952 (16)	
H37A	0.0859	0.8509	0.5024	0.114*	
H37B	0.1453	0.9125	0.5957	0.114*	
C38	0.2490 (8)	0.9569 (4)	0.4890 (5)	0.130 (2)	
H38A	0.3414	0.9881	0.5271	0.195*	
H38B	0.1694	1.0045	0.4730	0.195*	
H38C	0.2804	0.9269	0.4336	0.195*	
N1	0.8901 (4)	0.0114 (2)	1.1586 (2)	0.0562 (8)	
N2	0.3601 (4)	0.4957 (2)	0.7446 (2)	0.0557 (8)	
O1	0.4801 (4)	0.0199 (2)	0.7486 (2)	0.0933 (11)	
O2	0.9125 (4)	0.4760 (2)	1.1007 (2)	0.0861 (10)	
S1	0.79048 (15)	0.19795 (7)	1.06651 (8)	0.0734 (4)	
S2	0.45941 (13)	0.30926 (7)	0.83359 (7)	0.0617 (3)	

### Atomic displacement parameters (Å^2^)

**Table:**

	*U*^11^	*U*^22^	*U*^33^	*U*^12^	*U*^13^	*U*^23^
C1	0.053 (2)	0.050 (2)	0.048 (2)	0.0123 (17)	0.0076 (18)	0.0108 (17)
C2	0.064 (3)	0.048 (2)	0.071 (3)	0.0074 (18)	0.003 (2)	0.013 (2)
C3	0.067 (3)	0.050 (2)	0.069 (3)	0.0088 (19)	−0.001 (2)	0.000 (2)
C4	0.054 (2)	0.057 (2)	0.061 (3)	0.0105 (18)	0.007 (2)	0.004 (2)
C5	0.061 (2)	0.058 (2)	0.052 (2)	0.0141 (19)	0.009 (2)	0.0121 (19)
C6	0.060 (2)	0.048 (2)	0.062 (3)	0.0103 (17)	0.013 (2)	0.0098 (19)
C7	0.061 (3)	0.073 (3)	0.071 (3)	0.009 (2)	0.002 (2)	−0.002 (2)
C8	0.061 (2)	0.051 (2)	0.051 (2)	0.0096 (18)	0.0128 (19)	0.0030 (18)
C9	0.075 (3)	0.056 (2)	0.062 (3)	0.002 (2)	0.018 (2)	0.002 (2)
C10	0.073 (3)	0.074 (3)	0.061 (3)	−0.006 (2)	0.010 (2)	−0.011 (2)
C11	0.066 (3)	0.089 (3)	0.063 (3)	0.007 (2)	0.001 (2)	−0.006 (3)
C12	0.070 (3)	0.072 (3)	0.045 (2)	0.009 (2)	0.005 (2)	0.006 (2)
C13	0.057 (2)	0.057 (2)	0.044 (2)	0.0081 (18)	0.0135 (18)	0.0017 (18)
C14	0.068 (3)	0.059 (2)	0.047 (2)	0.0095 (19)	0.0128 (19)	0.0161 (18)
C15	0.067 (3)	0.061 (2)	0.060 (3)	0.012 (2)	0.009 (2)	0.019 (2)
C16	0.063 (3)	0.063 (3)	0.074 (3)	0.003 (2)	0.010 (2)	0.020 (2)
C17	0.061 (3)	0.068 (3)	0.096 (4)	0.004 (2)	−0.001 (2)	0.020 (2)
C18	0.096 (4)	0.070 (3)	0.173 (6)	0.009 (3)	0.020 (4)	0.045 (4)
C19	0.144 (6)	0.135 (5)	0.194 (8)	0.033 (5)	0.035 (6)	0.094 (5)
C20	0.061 (2)	0.055 (2)	0.051 (2)	0.0059 (18)	0.011 (2)	0.0107 (19)
C21	0.062 (2)	0.051 (2)	0.059 (3)	0.0127 (18)	0.019 (2)	0.0158 (19)
C22	0.053 (2)	0.053 (2)	0.050 (2)	0.0100 (17)	0.0137 (19)	0.0118 (18)
C23	0.056 (2)	0.050 (2)	0.052 (2)	0.0131 (17)	0.0175 (19)	0.0101 (18)
C24	0.076 (3)	0.046 (2)	0.057 (3)	0.0092 (19)	0.009 (2)	0.0109 (19)
C25	0.083 (3)	0.047 (2)	0.057 (3)	0.001 (2)	0.019 (2)	0.0037 (19)
C26	0.078 (3)	0.071 (3)	0.064 (3)	0.004 (2)	0.014 (2)	0.001 (2)
C27	0.054 (2)	0.055 (2)	0.053 (2)	−0.0017 (18)	0.0077 (18)	0.0091 (19)
C28	0.073 (3)	0.057 (2)	0.069 (3)	0.003 (2)	0.017 (2)	0.005 (2)
C29	0.103 (4)	0.078 (3)	0.065 (3)	0.001 (3)	0.017 (3)	−0.008 (3)
C30	0.100 (4)	0.093 (4)	0.051 (3)	0.001 (3)	0.001 (3)	−0.001 (3)
C31	0.083 (3)	0.074 (3)	0.055 (3)	0.012 (2)	0.004 (2)	0.015 (2)
C32	0.053 (2)	0.057 (2)	0.057 (3)	0.0070 (18)	0.0142 (19)	0.0111 (19)
C33	0.056 (2)	0.056 (2)	0.062 (3)	0.0153 (18)	0.0077 (19)	0.0164 (19)
C34	0.063 (3)	0.066 (3)	0.071 (3)	0.011 (2)	0.004 (2)	0.021 (2)
C35	0.075 (3)	0.073 (3)	0.066 (3)	0.019 (2)	0.012 (2)	0.023 (2)
C36	0.082 (3)	0.067 (3)	0.066 (3)	0.008 (2)	0.000 (2)	0.020 (2)
C37	0.116 (4)	0.085 (3)	0.088 (4)	0.030 (3)	0.013 (3)	0.031 (3)
C38	0.163 (7)	0.093 (4)	0.135 (6)	0.005 (4)	0.002 (5)	0.060 (4)
N1	0.067 (2)	0.0476 (18)	0.055 (2)	0.0072 (15)	0.0071 (17)	0.0155 (15)
N2	0.062 (2)	0.0523 (18)	0.054 (2)	0.0137 (15)	0.0071 (17)	0.0119 (15)
O1	0.108 (3)	0.092 (2)	0.074 (2)	0.028 (2)	−0.0122 (19)	0.0163 (19)
O2	0.086 (2)	0.096 (2)	0.073 (2)	0.0149 (18)	−0.0069 (18)	0.0213 (18)
S1	0.0895 (9)	0.0463 (6)	0.0770 (8)	0.0153 (5)	−0.0129 (6)	0.0065 (5)
S2	0.0761 (7)	0.0494 (6)	0.0601 (7)	−0.0003 (5)	0.0070 (5)	0.0159 (5)

### Geometric parameters (Å, °)

**Table:**

C1—N1	1.393 (5)	C20—C21	1.383 (5)
C1—C2	1.400 (5)	C20—C26	1.460 (6)
C1—C6	1.411 (5)	C21—C22	1.371 (5)
C2—C3	1.362 (5)	C21—H21	0.9300
C2—H2	0.9300	C22—C23	1.415 (5)
C3—C4	1.373 (5)	C22—S2	1.755 (4)
C3—H3	0.9300	C23—N2	1.397 (5)
C4—C5	1.380 (5)	C23—C24	1.399 (5)
C4—C7	1.464 (6)	C24—C25	1.372 (6)
C5—C6	1.367 (5)	C24—H24	0.9300
C5—H5	0.9300	C25—H25	0.9300
C6—S1	1.756 (4)	C26—O2	1.204 (5)
C7—O1	1.203 (5)	C26—H26	0.9300
C7—H7	0.9300	C27—C28	1.381 (5)
C8—C9	1.392 (5)	C27—C32	1.398 (5)
C8—C13	1.400 (5)	C27—S2	1.763 (4)
C8—S1	1.756 (4)	C28—C29	1.362 (6)
C9—C10	1.362 (6)	C28—H28	0.9300
C9—H9	0.9300	C29—C30	1.376 (6)
C10—C11	1.373 (6)	C29—H29	0.9300
C10—H10	0.9300	C30—C31	1.381 (6)
C11—C12	1.368 (6)	C30—H30	0.9300
C11—H11	0.9300	C31—C32	1.396 (6)
C12—C13	1.392 (5)	C31—H31	0.9300
C12—H12	0.9300	C32—N2	1.415 (5)
C13—N1	1.407 (5)	C33—N2	1.466 (4)
C14—N1	1.464 (4)	C33—C34	1.528 (5)
C14—C15	1.526 (5)	C33—H33A	0.9700
C14—H14A	0.9700	C33—H33B	0.9700
C14—H14B	0.9700	C34—C35	1.504 (5)
C15—C16	1.511 (5)	C34—H34A	0.9700
C15—H15A	0.9700	C34—H34B	0.9700
C15—H15B	0.9700	C35—C36	1.513 (6)
C16—C17	1.489 (5)	C35—H35A	0.9700
C16—H16A	0.9700	C35—H35B	0.9700
C16—H16B	0.9700	C36—C37	1.494 (6)
C17—C18	1.515 (6)	C36—H36A	0.9700
C17—H17A	0.9700	C36—H36B	0.9700
C17—H17B	0.9700	C37—C38	1.494 (7)
C18—C19	1.389 (7)	C37—H37A	0.9700
C18—H18A	0.9700	C37—H37B	0.9700
C18—H18B	0.9700	C38—H38A	0.9600
C19—H19A	0.9600	C38—H38B	0.9600
C19—H19B	0.9600	C38—H38C	0.9600
C19—H19C	0.9600	S1—S2	4.4392 (15)
C20—C25	1.378 (5)		
			
N1—C1—C2	122.1 (3)	C21—C22—C23	120.4 (3)
N1—C1—C6	121.4 (3)	C21—C22—S2	119.3 (3)
C2—C1—C6	116.4 (4)	C23—C22—S2	120.1 (3)
C3—C2—C1	121.5 (4)	N2—C23—C24	122.7 (3)
C3—C2—H2	119.2	N2—C23—C22	120.6 (3)
C1—C2—H2	119.2	C24—C23—C22	116.7 (4)
C2—C3—C4	121.6 (4)	C25—C24—C23	121.4 (4)
C2—C3—H3	119.2	C25—C24—H24	119.3
C4—C3—H3	119.2	C23—C24—H24	119.3
C3—C4—C5	118.0 (4)	C24—C25—C20	121.7 (4)
C3—C4—C7	120.4 (4)	C24—C25—H25	119.2
C5—C4—C7	121.5 (4)	C20—C25—H25	119.2
C6—C5—C4	121.5 (4)	O2—C26—C20	125.3 (4)
C6—C5—H5	119.2	O2—C26—H26	117.4
C4—C5—H5	119.2	C20—C26—H26	117.4
C5—C6—C1	120.8 (4)	C28—C27—C32	121.1 (4)
C5—C6—S1	118.0 (3)	C28—C27—S2	119.0 (3)
C1—C6—S1	120.8 (3)	C32—C27—S2	119.7 (3)
O1—C7—C4	126.3 (4)	C29—C28—C27	120.8 (4)
O1—C7—H7	116.9	C29—C28—H28	119.6
C4—C7—H7	116.9	C27—C28—H28	119.6
C9—C8—C13	120.5 (4)	C28—C29—C30	119.1 (4)
C9—C8—S1	118.0 (3)	C28—C29—H29	120.5
C13—C8—S1	121.2 (3)	C30—C29—H29	120.5
C10—C9—C8	121.2 (4)	C29—C30—C31	121.2 (5)
C10—C9—H9	119.4	C29—C30—H30	119.4
C8—C9—H9	119.4	C31—C30—H30	119.4
C9—C10—C11	118.5 (4)	C30—C31—C32	120.4 (4)
C9—C10—H10	120.7	C30—C31—H31	119.8
C11—C10—H10	120.7	C32—C31—H31	119.8
C12—C11—C10	121.4 (4)	C31—C32—C27	117.4 (4)
C12—C11—H11	119.3	C31—C32—N2	121.1 (3)
C10—C11—H11	119.3	C27—C32—N2	121.5 (4)
C11—C12—C13	121.4 (4)	N2—C33—C34	117.0 (3)
C11—C12—H12	119.3	N2—C33—H33A	108.0
C13—C12—H12	119.3	C34—C33—H33A	108.0
C12—C13—C8	116.9 (4)	N2—C33—H33B	108.0
C12—C13—N1	122.0 (3)	C34—C33—H33B	108.0
C8—C13—N1	121.1 (3)	H33A—C33—H33B	107.3
N1—C14—C15	117.3 (3)	C35—C34—C33	110.9 (3)
N1—C14—H14A	108.0	C35—C34—H34A	109.4
C15—C14—H14A	108.0	C33—C34—H34A	109.5
N1—C14—H14B	108.0	C35—C34—H34B	109.5
C15—C14—H14B	108.0	C33—C34—H34B	109.5
H14A—C14—H14B	107.2	H34A—C34—H34B	108.0
C16—C15—C14	109.5 (3)	C34—C35—C36	115.0 (4)
C16—C15—H15A	109.8	C34—C35—H35A	108.5
C14—C15—H15A	109.8	C36—C35—H35A	108.5
C16—C15—H15B	109.8	C34—C35—H35B	108.5
C14—C15—H15B	109.8	C36—C35—H35B	108.5
H15A—C15—H15B	108.2	H35A—C35—H35B	107.5
C17—C16—C15	116.2 (4)	C37—C36—C35	114.4 (4)
C17—C16—H16A	108.2	C37—C36—H36A	108.7
C15—C16—H16A	108.2	C35—C36—H36A	108.6
C17—C16—H16B	108.2	C37—C36—H36B	108.7
C15—C16—H16B	108.2	C35—C36—H36B	108.7
H16A—C16—H16B	107.4	H36A—C36—H36B	107.6
C16—C17—C18	113.6 (4)	C38—C37—C36	114.0 (5)
C16—C17—H17A	108.9	C38—C37—H37A	108.7
C18—C17—H17A	108.9	C36—C37—H37A	108.7
C16—C17—H17B	108.8	C38—C37—H37B	108.7
C18—C17—H17B	108.8	C36—C37—H37B	108.8
H17A—C17—H17B	107.7	H37A—C37—H37B	107.6
C19—C18—C17	119.6 (5)	C37—C38—H38A	109.5
C19—C18—H18A	107.4	C37—C38—H38B	109.5
C17—C18—H18A	107.4	H38A—C38—H38B	109.5
C19—C18—H18B	107.5	C37—C38—H38C	109.5
C17—C18—H18B	107.4	H38A—C38—H38C	109.5
H18A—C18—H18B	107.0	H38B—C38—H38C	109.5
C18—C19—H19A	109.4	C1—N1—C13	122.9 (3)
C18—C19—H19B	109.5	C1—N1—C14	118.1 (3)
H19A—C19—H19B	109.5	C13—N1—C14	118.2 (3)
C18—C19—H19C	109.5	C23—N2—C32	121.6 (3)
H19A—C19—H19C	109.5	C23—N2—C33	118.3 (3)
H19B—C19—H19C	109.5	C32—N2—C33	119.0 (3)
C25—C20—C21	117.6 (4)	C8—S1—C6	100.72 (18)
C25—C20—C26	120.6 (4)	C8—S1—S2	162.13 (14)
C21—C20—C26	121.8 (4)	C6—S1—S2	90.88 (14)
C22—C21—C20	122.2 (3)	C27—S2—C22	99.68 (18)
C22—C21—H21	118.9	C27—S2—S1	151.06 (13)
C20—C21—H21	118.9	C22—S2—S1	88.34 (13)
			
N1—C1—C2—C3	175.9 (4)	C30—C31—C32—C27	1.1 (6)
C6—C1—C2—C3	−1.2 (6)	C30—C31—C32—N2	−178.5 (4)
C1—C2—C3—C4	2.0 (6)	C28—C27—C32—C31	1.4 (6)
C2—C3—C4—C5	−0.5 (6)	S2—C27—C32—C31	−173.4 (3)
C2—C3—C4—C7	−179.6 (4)	C28—C27—C32—N2	−179.0 (4)
C3—C4—C5—C6	−1.8 (6)	S2—C27—C32—N2	6.1 (5)
C7—C4—C5—C6	177.3 (4)	N2—C33—C34—C35	175.1 (4)
C4—C5—C6—C1	2.6 (6)	C33—C34—C35—C36	177.1 (4)
C4—C5—C6—S1	−170.7 (3)	C34—C35—C36—C37	−179.4 (4)
N1—C1—C6—C5	−178.2 (3)	C35—C36—C37—C38	178.2 (5)
C2—C1—C6—C5	−1.1 (5)	C2—C1—N1—C13	157.4 (4)
N1—C1—C6—S1	−5.0 (5)	C6—C1—N1—C13	−25.7 (5)
C2—C1—C6—S1	172.1 (3)	C2—C1—N1—C14	−12.5 (5)
C3—C4—C7—O1	−175.6 (4)	C6—C1—N1—C14	164.4 (3)
C5—C4—C7—O1	5.4 (7)	C12—C13—N1—C1	−155.8 (4)
C13—C8—C9—C10	−2.3 (6)	C8—C13—N1—C1	25.3 (5)
S1—C8—C9—C10	172.0 (3)	C12—C13—N1—C14	14.1 (5)
C8—C9—C10—C11	1.6 (6)	C8—C13—N1—C14	−164.8 (3)
C9—C10—C11—C12	0.2 (7)	C15—C14—N1—C1	85.4 (4)
C10—C11—C12—C13	−1.5 (6)	C15—C14—N1—C13	−85.0 (4)
C11—C12—C13—C8	0.8 (6)	C24—C23—N2—C32	152.8 (4)
C11—C12—C13—N1	−178.2 (4)	C22—C23—N2—C32	−27.4 (5)
C9—C8—C13—C12	1.0 (5)	C24—C23—N2—C33	−14.9 (5)
S1—C8—C13—C12	−173.1 (3)	C22—C23—N2—C33	164.9 (3)
C9—C8—C13—N1	−180.0 (3)	C31—C32—N2—C23	−151.2 (4)
S1—C8—C13—N1	5.9 (5)	C27—C32—N2—C23	29.2 (5)
N1—C14—C15—C16	−173.9 (3)	C31—C32—N2—C33	16.4 (5)
C14—C15—C16—C17	−174.2 (4)	C27—C32—N2—C33	−163.1 (3)
C15—C16—C17—C18	−178.5 (4)	C34—C33—N2—C23	83.5 (4)
C16—C17—C18—C19	−176.0 (6)	C34—C33—N2—C32	−84.5 (4)
C25—C20—C21—C22	−0.1 (6)	C9—C8—S1—C6	157.7 (3)
C26—C20—C21—C22	178.0 (4)	C13—C8—S1—C6	−28.0 (3)
C20—C21—C22—C23	2.8 (6)	C9—C8—S1—S2	28.0 (6)
C20—C21—C22—S2	−171.3 (3)	C13—C8—S1—S2	−157.7 (3)
C21—C22—C23—N2	176.6 (3)	C5—C6—S1—C8	−159.1 (3)
S2—C22—C23—N2	−9.3 (5)	C1—C6—S1—C8	27.6 (4)
C21—C22—C23—C24	−3.6 (5)	C5—C6—S1—S2	7.2 (3)
S2—C22—C23—C24	170.5 (3)	C1—C6—S1—S2	−166.1 (3)
N2—C23—C24—C25	−178.3 (4)	C28—C27—S2—C22	152.0 (3)
C22—C23—C24—C25	1.9 (6)	C32—C27—S2—C22	−33.1 (3)
C23—C24—C25—C20	0.7 (6)	C28—C27—S2—S1	47.6 (5)
C21—C20—C25—C24	−1.6 (6)	C32—C27—S2—S1	−137.4 (3)
C26—C20—C25—C24	−179.8 (4)	C21—C22—S2—C27	−151.1 (3)
C25—C20—C26—O2	176.4 (4)	C23—C22—S2—C27	34.8 (3)
C21—C20—C26—O2	−1.7 (7)	C21—C22—S2—S1	0.9 (3)
C32—C27—C28—C29	−2.5 (6)	C23—C22—S2—S1	−173.2 (3)
S2—C27—C28—C29	172.4 (4)	C8—S1—S2—C27	75.2 (5)
C27—C28—C29—C30	1.0 (7)	C6—S1—S2—C27	−55.7 (3)
C28—C29—C30—C31	1.6 (8)	C8—S1—S2—C22	−32.0 (4)
C29—C30—C31—C32	−2.6 (7)	C6—S1—S2—C22	−162.86 (17)

### Hydrogen-bond geometry (Å, °)

**Table:**

*D*—H···*A*	*D*—H	H···*A*	*D*···*A*	*D*—H···*A*
C28—H28···O1	0.93	2.54	3.454 (5)	168
C9—H9···O2	0.93	2.50	3.394 (5)	162
